# Supplementary material for: Eco-friendly fashion among generation Z: Mixed-methods study on price value image, customer fulfillment, and pro-environmental behavior
Source: PLoS One. 2022 Aug 16;17(8):e0272789. doi: 10.1371/journal.pone.0272789 (PMC9380922; doi:10.1371/journal.pone.0272789)
Supplement: S1 File — (PDF) [file pone.0272789.s001.pdf]

Eco-friendly Fashion Question Guide

Interview Goals

1. In Vietnam: How does gen Z define Sustainable and Eco-friendly fashion, have you bought it yet?
2. What factors influence buying Sustainable and Eco-friendly fashion (expected and actual). What factors determine the buying behavior and loyalty of users? Do they care about the environment?
3. What factors determine Gen Z to buy and recommend to others about Sustainable and Eco-friendly fashion
4. According to the interviewee, how to promote the consumption of Sustainable and Eco-friendly fashion

1. How does Vietnamese gen Z define eco-friendly fashion products?

- Have you ever heard of eco-friendly fashion products?
- If so, which channel did you get this information from?
- What is your definition of eco-friendly fashion products? Can you give some examples of fashion products that are eco-friendly according to the above definition?
- Have you ever purchased or used any environmentally-friendly fashion products?

2. Factors influencing purchase intention of eco-friendly fashion products? (hypothetically and actually)

2.1 If the interviewee has purchased eco-friendly fashion products:

- Why did you decide to buy that product?
- Do you have any difficulties in purchasing eco-friendly fashion products? If so, what did you do to solve them?
- When purchasing these products, which other factors would you consider?
- Which of the above factors is most important to you? Why?
- How did the mentioned factors affect your purchase intention?

2.2 If the interviewee has heard of eco-friendly fashion products but not purchased:

- Why don't you choose to use eco-friendly fashion products? Are there any difficulties that prevent your purchase intentions?
- When buying these products, what factors will you consider?
- Which factors do you think are the most important determinant of purchase intention?

2.3. If the interviewee does not know eco-friendly fashion products

- When purchasing these products, what factors will you consider? Can you give us the reasons why?
- Which factors do you think are the most important determinant of purchase intention?
- What do you expect from an environmentally friendly fashion product?

3. Factors influencing repurchase intention and recommendation of eco-friendly fashion products?

- What are factors influencing your repurchase intention of eco-friendly fashion products? Which is the deciding factor?
- What are the factors determining your repurchase intention of an eco-friendly fashion store? Which is the deciding factor?
- When using a favorite fashion product, do you often recommend to your friends or family?
- If your friends did not use eco-friendly fashion products, how would you convince them to purchase eco-friendly fashion products?
- What is the deciding factor?
- If necessary, would you recommend an environmentally friendly fashion product or an environmentally unfriendly fashion product to your relatives and friends? Can you give us the reasons why?

4. Suggestions to encourage the popularity of eco-friendly fashion products?

Can you suggest some ways to encourage more people to use eco-friendly fashion?
